# Attitudes, practices, and zoonoses awareness of community members involved in the bushmeat trade near Murchison Falls National Park, northern Uganda

**DOI:** 10.1371/journal.pone.0239599

**Published:** 2020-09-28

**Authors:** BreeAnna M. Dell, Marcy J. Souza, Adam S. Willcox

**Affiliations:** 1 Department of Biomedical & Diagnostic Sciences, University of Tennessee, Knoxville, Tennessee, United States of America; 2 Department of Forestry, Wildlife and Fisheries, University of Tennessee, Knoxville, Tennessee, United States of America; University of Pretoria, SOUTH AFRICA

## Abstract

The harvest of bushmeat is widespread in the tropics and sub-tropics. Often in these communities, there is a dependence on bushmeat for both food security and basic income. Despite the importance of bushmeat for households worldwide, the practice raises concern for transmission of zoonotic pathogens through hunting, food preparation, and consumption. In Uganda, harvest of wildlife is illegal, but bushmeat hunting, is commonplace. We interviewed 292 women who cook for their households and 180 self-identified hunters from 21 villages bordering Murchison Falls National Park in Uganda to gain insights into bushmeat preferences, opportunity for zoonotic pathogen transmission, and awareness of common wildlife-associated zoonoses. Both hunters and women who cook considered primates to be the most likely wildlife species to carry diseases humans can catch. Among common zoonotic pathogens, the greatest proportions of women who cook and hunters believed that pathogens causing stomach ache or diarrhea and monkeypox can be transmitted by wildlife. Neither women who cook nor hunters report being frequently injury during cooking, butchering, or hunting, and few report taking precautions while handling bushmeat. The majority of women who cook believe that hunters and dealers never or rarely disguise primate meat as another kind of meat in market, while the majority of hunters report that they usually disguise primate meat as another kind of meat. These data play a crucial role in our understanding of potential for exposure to and infection with zoonotic pathogens in the bushmeat trade. Expanding our knowledge of awareness, perceptions and risks enables us to identify opportunities to mitigate infections and injury risk and promote safe handling practices.

## 1. Introduction

The hunting and consumption of bushmeat is a widespread practice in tropical and subtropical ecosystems, often to provide food security and supplement basic income for participating households. Estimates for households dependent on bushmeat as a meat source surpass 150 million in the Global South [1]. In recent studies, 39% of surveyed households in 24 countries reported hunting bushmeat. Of the households reporting having hunted bushmeat, they further reported that 89% of the bushmeat harvest was directly applied to dietary needs [1,2]. Additionally, bushmeat hunting tends to be most prevalent in areas with greater biodiversity indices, which frequently align with regions experiencing higher poverty and food insecurity [3–5]. In Uganda alone, over 71% of households reported having participated at some point in bushmeat harvest and/or consumption [2]. The widespread dependence of populations on bushmeat for nutritional and financial security raises concern for the sustainability of hunting practices for wildlife populations where bushmeat harvest is prevalent and for the risk of exposure of hunters and consumers to emerging, reemerging, and endemic diseases during hunting, preparation, and consumption [6–8].

Human contact with wildlife is a major pathway for emerging and endemic infectious diseases, with 62% of all newly emerging infectious diseases being zoonotic and over 70% of those zoonoses implicating wildlife reservoirs [9]. The bushmeat trade presents numerous routes of opportunity for transmission of zoonotic pathogens, including airborne and blood-borne during hunting and the butchering of carcasses, as well as foodborne risks associated with preparation and consumption. Consumption-related risks are especially relevant in areas where there is suboptimal storage of meat in the consumer chain, allowing proliferation of bacterial pathogens [10–12]. Moreover, information about the effects of hunting and associated diseases remain limited largely due to poor healthcare access and reporting in many regions where bushmeat hunting and consumption is common. Recent epidemics have instilled zoonotic diseases into the global consciousness following large-scale and highly publicized outbreaks such as the 2015 and ongoing Ebola virus epidemics and the recent COVID-19 pandemic; each of these infectious agents originated from contact with wildlife species [13–17]. Less highly publicized, but arguably more pervasive in many local communities is the presence of endemic zoonotic bacterial pathogens in hunted wildlife such as *Shigella*, *Campylobacter*, *Listeria*, *Pseudomonas*, *Staphylococcus*, *Salmonella*, *Shigella*, *E*. *coli*, and *Brucella* among others [6,11,18–22]. Diarrheal and other foodborne illnesses are still a significant cause of mortality, disability, and economic loss in many countries [23–25].

An additional concern is that pathogens from hunted wildlife may also be brought into contact with domestic animal species. African swine fever, avian influenza, rabies, anthrax, tuberculosis, brucellosis, and Rift Valley Fever are among some of the most well-studied diseases that can be transmitted from wildlife to livestock with contact. These infections result in poor animal health outcomes, resulting in negative impacts to farmer livelihoods, and may continue to circulate between livestock and wildlife through these animals’ contact networks [26–28]. Many of these multi-host animal pathogens may also spillover from livestock to cause sporadic cases or outbreaks of disease in humans [18,29–31]. Risk for human cases of these diseases may increase substantially in subsistence farm settings, where extensive contact with domestic animals and handling of animal products occurs daily.

Despite increasing interest in wildlife-acquired zoonoses, much of the information we have on the prevalence and practice of bushmeat in communities comes from geographically-limited surveys of hunters and small-scale studies reporting market observations, which give limited insight to the bushmeat markets in other communities, even within the same region or country [32]. Bushmeat serves as a vital resource in many rural lower-income regions of sub-Saharan Africa, but more research on the prevalence and drivers of the bushmeat trade has been conducted in West Africa and Central Africa than in East Africa. Estimates attribute nearly 90% of consumed animal protein in West and Central Africa to bushmeat, with daily wild meat consumption ranging from 0.008kg/day in Libreville, Gabon to up to 0.22kg/day in Campo, Cameroon [33–35]. The widespread dependence of households on bushmeat is generally accepted as fact but only sporadically documented, with data particularly lacking in East Africa. Because the cultural, legal, and sociopolitical differences among communities engaged in bushmeat trade are distinct, there are gaps in our understanding of what drives the bushmeat trade. This limitation reduces our ability to understand how to effectively mitigate the associated risks of bushmeat hunting and consumption.

In Uganda, hunting of all wildlife species by citizens is illegal and a punishable offence under the Uganda Wildlife Act of 2000 [36]. There is exception to this if a vermin species depredates crops on private land, in which case the animal can be disposed under the permission and supervision of the Uganda Wildlife Authority (UWA) [37–42]. There are currently three recognized vermin species: bushpigs (*Potamochoerus larvatus*), vervet monkeys (*Chlorocebus pygerythrus*), and olive baboons (*Papio anubis*) [36]. Despite legal restrictions on hunting wildlife, bushmeat harvest is widespread and culturally accepted [43,44]. The illegal nature of the practice has resulted in a covert market with person-to-person exchanges rather than legal open markets. Furthermore, in initial communications with Ugandan collaborators on this project, the concept of “species deception” in market emerged, in which bushmeat is sold to consumers by either hunters or dealers as a different species than the true species. It has been demonstrated that nearly 30% of bushmeat sold in these same communities are misrepresented as another species of bushmeat [45]. This practice adds an additional degree of risk to the bushmeat chain, as certain species of wildlife, such as primates, and bats are more often implicated as reservoirs for zoonotic diseases of consequence than species like warthog or antelope, which are more culturally desirable to consume and historically considered to be lower risk animals for zoonotic spillover events [46,47]. However, despite the established belief that bats and rodents hold a greater importance for spillover of zoonotic viral pathogens, recent research suggests a more host-neutral explanation for spillover than previously thought, which may have implications for handling precautions and practices based upon this belief [48].

In this paper, we present bushmeat hunting and handling survey data collected from hunters and women who cook for their households (hereafter noted as “cooks”) in 21 communities adjacent to protected areas in northern Uganda. Cooks and hunters were chosen as they represent the population subsets in greatest contact with bushmeat and most in control of implementing practices that might minimize exposure to zoonotic pathogens. Our research objectives for this study were to elucidate drivers of participation in the bushmeat trade, gain insight into hunting practices and bushmeat preparation in our study area, and to establish an understanding of the level of local knowledge of zoonotic disease risk from participation in these activities. These data serve as an important resource to begin to understand this ubiquitous, but clandestine, practice and to inform policy and community engagement to prevent both emerging and endemic zoonotic illnesses in these communities. Furthermore, insights gained from these data should be used to empower local community members, district leaders and public health stakeholders to increase safety measures that prevent and reduce the incidence of zoonotic infections resulting from contact with bushmeat.

## 2. Methods

### 2.1 Study area

The Murchison Falls Conservation Area (MFCA) is Uganda’s largest and oldest continuous protected area, comprised of the 3,893 km^2^ Murchison Falls National Park (MNFP) to the north, the 748 km^2^ Bugungu Wildlife Reserve to the southwest, and the 720 km^2^ Karuma Falls Wildlife Reserve to the southeast. The park was initially founded in 1926 as a game reserve to preserve the savannah, forests, and Murchison Falls, a major tourist attraction for its high flow rate and beauty, and then gazetted as a national park in 1952 following the National Parks Act [49]. The existing protected area sits at the northern terminus of the Albertine Rift and is notable for its high biodiversity of both mammalian and avian species [50]. The MFCA is managed and operated by the Uganda Wildlife Authority and is used primarily for conservation and ecotourism. MFNP is the second most visited national park in the country with 75,360 visitors (30.7% of all national park visits) reported by the Ministry of Tourism, Wildlife, and Antiquities in 2016. Of these visitors, 29,868 are non-residents and foreigners. Estimated revenue from entrance to all Ugandan protected areas and related recreational activities for UWA is UGX 92,628,231,456 [51]. Revenue sharing at 20% of tourism to MFNP resulted in disbursement of UGX 8,421,310,000 (USD 2,285,945.79) to the surrounding communities for livelihood projects “geared towards management of human wildlife conflicts, livelihood improvement, and common good in the frontline parishes” from 2012–2018 and UGX 10,290,101,500 (USD 2,793,225.07) total since 2005 [51]. Projects funded by revenue sharing in bordering MFNP have included classroom block construction and school staff accommodation, health unit construction, sanitation projects, and livestock-based income-generating activities (such as goat, poultry, and rabbit rearing and bee-keeping) [52].

**Fig 1:**
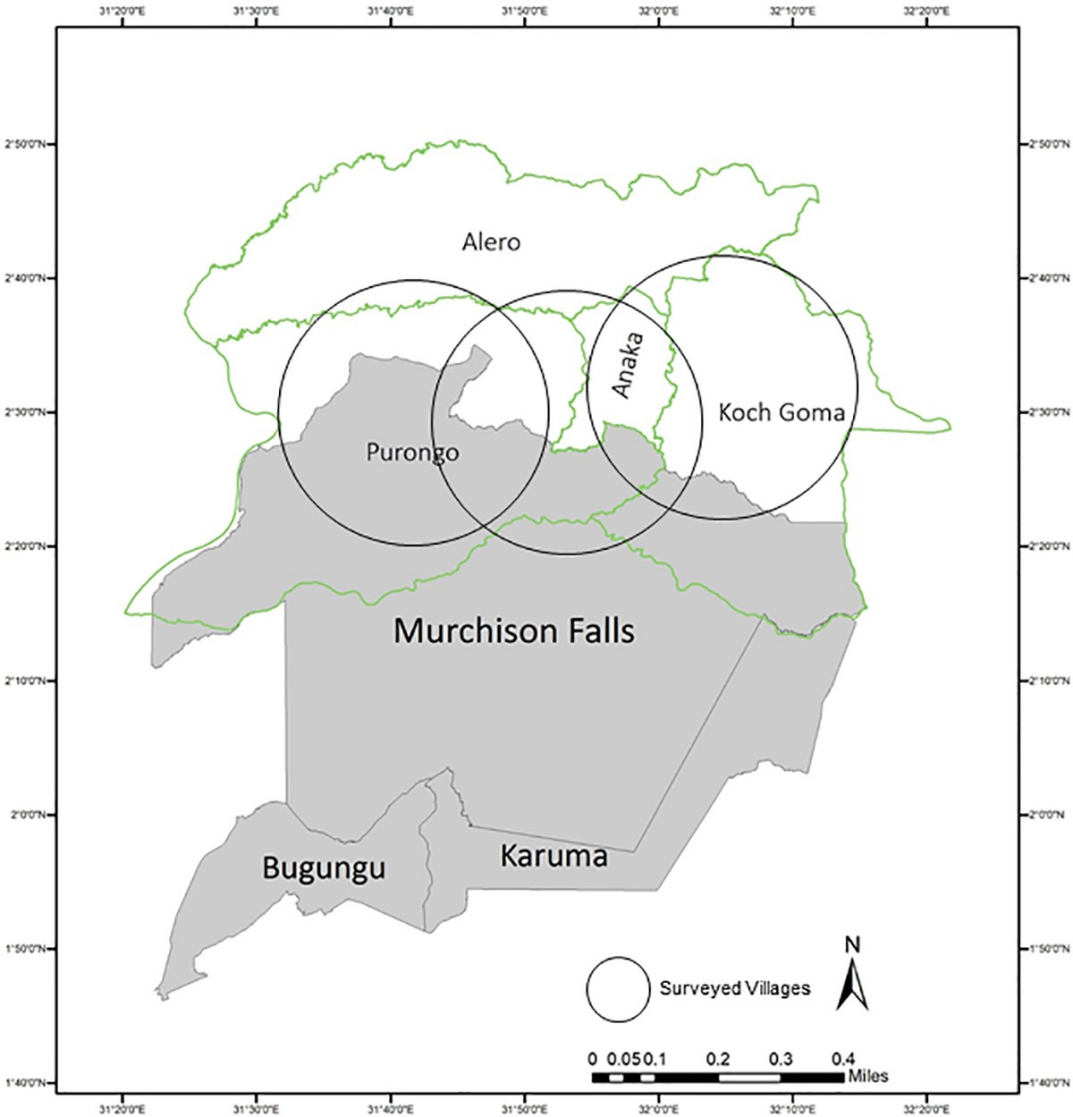
Map of the Murchison Falls Conservation Area (Bugungu Wildlife Preserve, Karuma Falls Wildlife Preserve, and Murchison Falls National Park) and the northern adjacent district, Nwoya. Nwoya district boundaries are delineated by the green borders and divided into its four subdistricts (Koch Goma, Anaka, Alero, and Purongo). Black circles indicate general undisclosed locations where interviews were conducted with hunters and cooks, 2016–2017.

Human population density in the areas surrounding MFNP has increased from an estimated 18 individuals/km^2^ in 1959 to 111 individuals/km^2^ reported on the 2014 census [53]. Our study was conducted in villages in Nwoya district in northern Uganda. Nwoya district is composed of 4 sub-counties, Purongo, Anaka, Alero, and Koch Goma, and forms the northernmost border of Murchison Falls Conservation Area (MFCA) The population of Nwoya district in the 2014 census was 133,506, with a projected population in 2019 of 214,200 [54]. Nwoya district reports a population density of 23 individuals/km^2^ and an average household size of 5 individuals [54]. A map of the study area can be seen in Fig 1.

### 2.2 Survey design

Our survey was constructed in cooperation with our partners at Makerere University and our governmental partner, the private secretary in charge of veterinary affairs in the State House of Uganda. The survey instrument was designed to gain insight to the attitudes, practices, perceived risk, and preferences surrounding the bushmeat trade in the greater MFNP region so that appropriate educational and disease prevention measures could be implemented with increased efficacy. The survey contained questions about meat preference, perceived risk of injury and disease during activities involving bushmeat, knowledge of zoonotic diseases, availability of species in market, and demographic information (S1 and S2 Files). Questions were presented in a variety of formats, including multiple choice, ordered response, free response, and battery-type statements with Likert-type response choices.

We constructed the survey in English and translated it into Acholi. The Acholi survey was then back-translated to ensure clarity and understanding of survey items. The survey instrument was developed and sent to Ugandan academic experts and colleagues to confirm content validity. We pilot tested the hunter survey instrument using cognitive interviews with three Acholi-speaking hunters [55]. We pilot tested the female cook survey instrument using a group cognitive interview of seven female Acholi-speaking community members and separate cognitive interviews with three Ugandan academic colleagues to ensure questions were appropriate and easily understood. If a question contained language that was not easily understood or conveyed a meaning that was not intended, the question was rewritten and rechecked with pilot group members before being deployed in the field. All survey materials and research procedures were approved by the University of Tennessee’s Office of Research and Engagement’s Institutional Review Board (protocol number UTK IRB-16-03109-XM & UTK IRB 16-3158-XM) and the Uganda National Council for Science and Technology (research registration number HS 3013). Site-specific permissions were secured through oral consent by local leaders. Local field staff obtained oral informed consent for voluntary individual participation. The iSurvey iPad application (Harvestyourdata 2016 & 2017) was used to administer the questionnaire in the field and store response data locally on the tablets and then uploaded to the program’s data cloud each evening.

All research methods and informed consent procedures were reviewed and approved by the University of Tennessee’s Institutional Review Board (UTK IRB-16-03109-XM & UTK IRB 16-3158-XM) and Uganda National Council for Science and Technology (research registration number HS 3013). Interested potential participants were read the approved informed consent statement and verbally agreed to participate or declined and the survey ended at that time and a new form was opened. An enumerator button was present on the front page of the iPad which had to be clicked following verbal consent from the respondent before the survey could be started. Informed consent for participation was obtained using these methods for the following reasons: the study was anonymous and given exempt review status by the University of Tennessee, the clandestine nature of the bushmeat market in Uganda would likely have negatively affected response/refusal rates, and administration of survey items was performed using an iPad and therefore no paper was used. Printed paper consent forms were made available upon request.

### 2.3 On-site interviews

Hunter interviews were conducted over a two-week period in July 2016 in 10 villages in Nwoya district with individuals who self-identified as having hunted wildlife in MFNP. We selected villages based on their proximity and accessibility to MFNP and expected participation in the bushmeat trade as identified by our local collaborators. Initial hunter respondents in each village were identified by our community liaisons. The liaisons for this research period were two men who were local community members with a demonstrated history of involvement in scientific research with collaborators at Makerere University, fluency in Acholi, and knowledge and familiarity with local hunters and bushmeat markets. We obtained subsequent interviews through word-of-mouth among hunters and through a snowball sampling technique in which initial respondents recruited other hunters [56]. This method was utilized since illegal hunting is a sensitive topic with potential to carry penalties to those involved if participants were implicated. Moreover, this method is used routinely in studies focused on populations that may be difficult to identify [57]. Respondents were assured anonymity and all respondents participated voluntarily and were not incentivized to participate in this study with gifts or monetary payment.

Interviews with female cooks were conducted over a 3-week period in July 2017 in 21 villages and communities in Nwoya district. The same 10 villages as in 2016, as well as additional sub-communities of the original villages in which women worked, were sampled. We attempted to interview every woman involved in household food preparation in each village included in the study area. One to four days before interviewing in a village, our community liaison traveled to that village to describe our study to women living in the community and arrange a time at which willing cooks could gather for interviews. Interviews were conducted one-on-one in Acholi, except in instances when participants were uncomfortable responding to questionnaires alone. In these cases, groups of two to three women would be asked questionnaire items in proximity and each individual participant’s response would be recorded separately. In this case, printed paper questionnaires were used to record responses from each respondent and later entered into iSurvey by researchers. All paper survey results were entered manually the same day interviews were conducted and checked for data entry errors. As with hunter surveys, all cooks interviewed participated voluntarily and were not incentivized to participate in the study with gifts or monetary payment.

### 2.4 Statistical analysis

All statistical analyses were performed using IBM SPSS Statistics 25. We used descriptive statistics to summarize survey data. Comparisons of proportions between hunters and cooks were assessed with z-tests with Bonferroni correction. Statistical significance was concluded at *P* ≤ 0.05 for all tests. Constructs of hunters’ and cooks’ perceived risk of zoonotic diseases through contact with bushmeat were assessed using principal components factor analysis with a Varimax rotation [58]. Factors were extracted based on Eigenvalues greater than 1 and confirmed via Monte-Carlo parallel analysis [59–62]. Threshold for retention of variables in final analysis was 0.5. Variables below this were removed and the factor analysis was re-run. Cronbach’s α was used to assess the final extracted factor reliability [63].

## 3. Results

### 3.1 Socio-demographic information

Demographic information for cooks and hunters is summarized in Table 1. We interviewed 180 self-identified hunters in 10 communities adjacent to MFNP. Hunters were generally younger adults ($\bar{x}$ ± SD; 33 years±10.9), ranging from 18 years to 74 years old. The majority of hunters reported having lived in the community since birth (n = 110; 60.8%). Most hunters reported primary school as their highest level of education (n = 137; 76.1%), and most were married (n = 158; 87.8%). An overwhelming majority of hunters reported their primary occupation as farmer (n = 167; 92.8%), while only three respondents (1.7%) identified their primary occupation as hunter.

**Table 1:** Demographic information of interviewed cooks and hunters from communities in Nwoya District, Uganda 2016–2017.

Hunters (n = 180)		Cooks (n = 292)	
**Age** ($\bar{x}$**±SD)**	33.0 ± 11.0	**Age** ($\bar{x}$**±SD)**	37.3 ± 14.4
**Marital Status**		**Marital Status**	
Married	158 (87.3%)	Married	199 (66.1%)
Divorced	7 (3.9%)	Divorced	23 (7.6%)
Widowed	2 (1.1%)	Widowed	58 (19.3%)
Never married	14 (7.7%)	Never married	21 (7%)
**Education Level**		**Education Level**	
Technical/trade school	1 (0.6%)	Technical/trade school	4 (1.3%)
Secondary school	38 (21.0%)	Secondary school	36 (12.0%)
Primary school	138 (76.2%)	Primary school	183 (60.8%)
College or university	4 (2.2%)	Informal/no schooling	78 (25.9%)
**Years Lived in Community**		**Years Lived Community**	
1–5 years	37 (20.6%)	1–5 years	103 (35.3%)
6–10 years	20 (11.1%)	6–10 years	85 (29.1%)
11–20 years	13 (7.2%)	11–20 years	42 (14.4%)
21+ years	109 (60.6%)	21+ years	62 (21.2%)
**Primary Occupation**		**Primary Occupation**	
Farmer	167 (92.8%)	Farmer	220 (75.3%)
Businessman	3 (1.7%)	Vendor	28 (9.6%)
Hunter	3 (1.7%)	Businesswoman	14 (4.8%)
Motorcycle taxi	3 (1.7%)	Food service worker	8 (2.7%)
Quarry worker	1 (0.6%)	No occupation	7 (2.4%)
Mechanic	1 (0.6%)	Tailor	5 (1.7%)
Teacher	1 (0.6%)	Hairdresser	5 (1.7%)
Surveyor	1 (0.6%)	Hotel owner	2 (0.7%)
		Childcare giver	1 (0.3%)
		Teacher	1 (0.3%)
		Savings group chair	1 (0.3%)

We interviewed 292 women who cook for their households from 21 communities. The mean age of cooks was 37 (±14.2) years, ranging from 18 years to 81 years old. Unlike hunters, most cooks did not live in the community since birth, with only 22 (7.5%) of respondents being born in the respective study villages. Cooks’ mean length of time spent living in the community was 13 (±14.1) years, ranging from one year to 70 years. The majority of cooks reported primary school as their highest level of education (n = 175; 59.9%, and most were married (n = 193; 66.1%). The most common primary occupation among cooks was farmer (n = 222; 76.0%); however, more than one primary occupation was reported by twenty-six respondents (8.9%).

### 3.2 Hunting techniques and practices

Hunters indicated that the African buffalo (*Syncerus caffer caffer*) is the most dangerous wild animal to hunt (44.2%, n = 80) and the most dangerous to trap (48.6%, n = 88). Hunters used spears to hunt more than once per week (1.40 ±1.06), and dogs (2.04±1.50), wire snares (3.13±2.70), and sticks/clubs (2.36±1.90) less frequently, where 1 = nearly every day, 2 = at least 3 times per week, 3 = once a week, 4 = several times per month, 5 = several times per year, and 6 = never. When asked about the safety of hunting techniques, hunters perceived bow hunting as the most dangerous hunting technique (3.43±0.99), followed by trapping (2.63±0.99), spear hunting (2.47±0.87), and hunting with dogs (2.46±0.82), where 1 = very safe, 2 = safe, 3 = neither safe nor dangerous, 4 = dangerous and 5 = very dangerous. Although most hunters did not report being frequently wounded, they reported being wounded most often during butchering (3.09±1.08), followed by trapping (2.2±1.05), spear hunting (1.93±0.98), then hunting with dogs (1.77±1.08), where 1 = never, 2 = rarely, 3 = sometimes, 4 = frequently, 5 = every time. Fifty-eight percent (n = 105) of hunters reported having harvested, hunted, or trapped baboons or monkeys (69.1%, n = 125) and bats (63.5%, n = 115). Only 5% (n = 9) of hunters reported taking any kind of safety precaution when hunting, trapping, or handling bushmeat. The most frequently reported precaution taken was to “leave bones in bush” (n = 4). One respondent described wearing plastic bags on his hands as gloves.

### 3.3 Food preparation practices

A greater proportion of cooks reported taking precautions when preparing domestic meats (n = 79; 27.1%) compared to when preparing bushmeat (n = 68; 23.3%). Most cooks reported sometimes being wounded while preparing or cooking meat (n = 163; 55.8%), then rarely (n = 67; 22.9%), never (n = 45; 15.4%), frequently (n = 16; 5,5%), and usually (n = 1; 0.3%). The mean number of adults cooked for on a daily basis was 3.6 (SD ±2.2), ranging from one to 16 adults per single respondent; mean number of children cooked for on a daily basis was 4.9 (SD ±3.3), ranging from one to 40 children per single respondent.

### 3.4 Comparative hunter and cook results

#### Meat preference and market value

**Fig 2:**
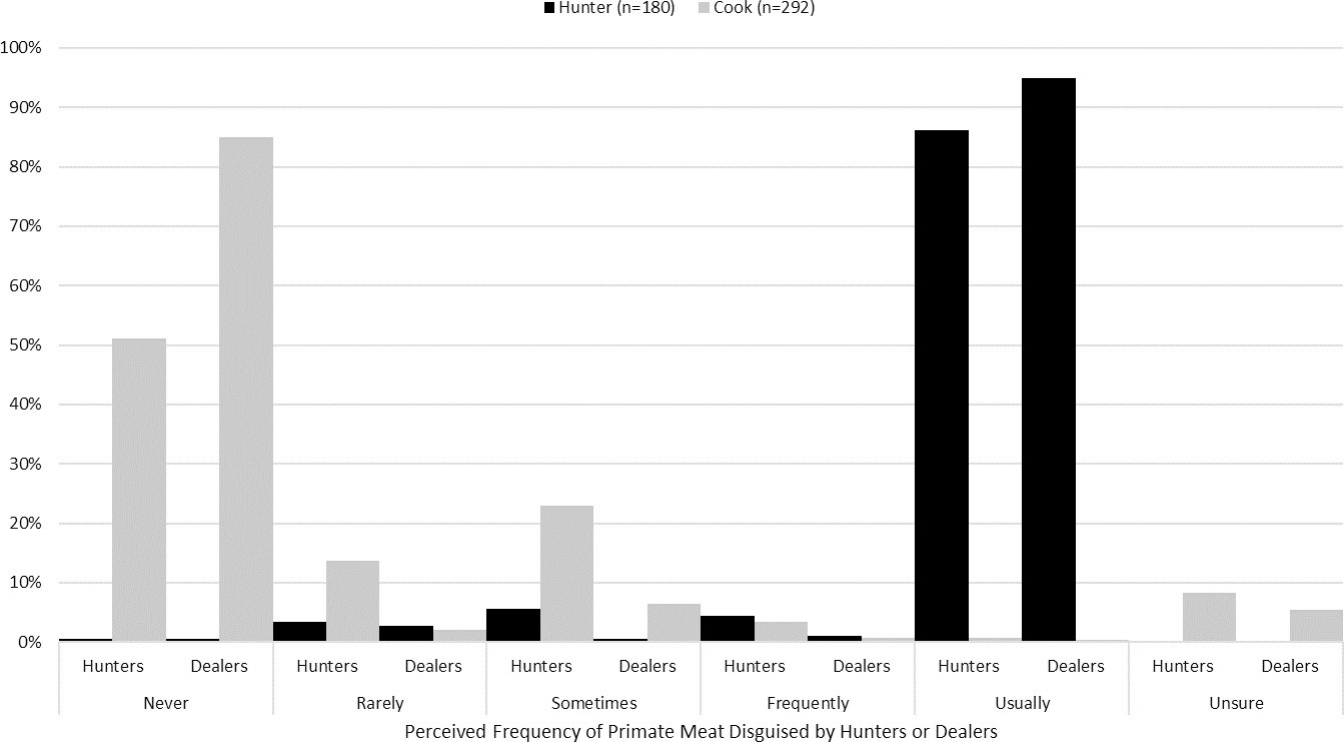
Cook and hunter responses to which type of meat they most prefer to eat from among wild and domestic choices in Nwoya district, Uganda, 2016–2017.

Meat preference data are displayed in Fig 2. Overall, hunters preferred the taste of bushmeat over domestic meats. However, on an animal-by-animal basis, hunters reported that the most delicious animal was domestic chicken (n = 31, 17.2%), followed by antelope and warthog (each n = 28, 15.6%), hippopotamus (n = 22, 12.2%), and goat and edible bush rat (each n = 21, 11.7%). Antelope was the most frequently reported most delicious wild meat when only wild meat options were listed (n = 49, 27.21%,). Most (n = 95, 52.8%) hunters preferred to eat meat from either wildlife or domestic species overall compared to either fish (n = 27, 15.0%) or beans/vegetables (n = 58, 32.2%).

Generally, cooks preferred the taste of domestic meats to bushmeat. Chicken (n = 116; 38.5%) was ranked the most delicious meat, followed by goat (n = 89; 29.6%), beef (n = 53; 17.6%), warthog (n = 9; 3.3%), and pork (n = 9; 3%). Cooks also selected domestic meat choices as the most nutritious, indicating chicken (n = 146; 48.5%), goat (n = 77; 25.6%), and beef (n = 33; 11%) as the most nutritious meats. Cooks identified bushmeat (4.05±0.9) as being more expensive in market than domestic meat choices (3.0±1.006), where 1 = very cheap, 2 = cheap, 3 = neither cheap nor expensive, 4 expensive, and 5 = very expensive. The majority of cooks reported that they “never” knowingly consumed baboons (n = 270; 90%), monkey species (n = 271; 90%), chimpanzees (n = 279; 92.7%), or bats (n = 279; 92.7%).

#### Disease knowledge/food safety

**Fig 3:**
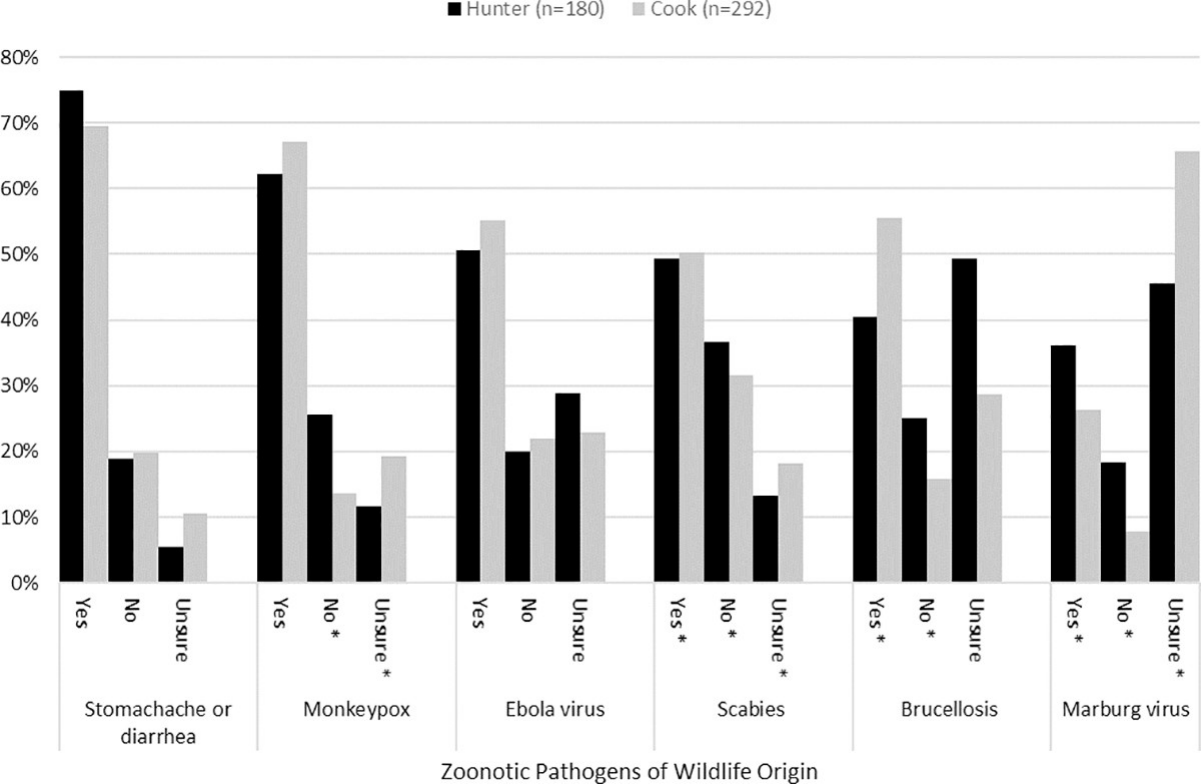
Cook and hunter responses to whether they believe wildlife species can carry select zoonotic diseases, Nwoya district, Uganda, 2016–2017. Proportions of cooks and hunter participants sharing for response categories denoted by * differ significantly from each other at P ≤ 0.05.

When queried about knowledge of major diseases being carried and spread to humans by wildlife, hunter responses were varied. Stomach ache and other diarrheal illnesses were most acknowledged for their zoonotic potential at 74.6% (n = 135) followed by 62.2% (n = 112) for monkeypox. Marburg virus (35.9%; n = 65) and brucellosis (40.3%; n = 73) had the least zoonotic potential awareness. Cook responses to this question were similar to hunters’, with the most awareness for stomach ache and diarrheal illness (69.5%; n = 203) and monkeypox (67.1%; n = 196) and the least for Marburg virus (26.4%; n = 77). Cook and hunter response proportions differed significantly from each other for Marburg virus, monkeypox, brucellosis, and scabies, but not for Ebola virus or stomach ache and diarrheal illness. These data are summarized in Fig 3. Furthermore, hunters indicated that they believed wildlife were most likely to carry diseases livestock could catch (3.55±1.18), followed by people (3.5±1.19), and least likely to carry disease that hunting dogs could catch (3.37±1.3), where 1 = very unlikely, 2 = unlikely, 3 = neither unlikely nor likely, 4 = likely, and 5 = very likely.

Cooks considered domestic meat consumption (cow, pig, chicken, goat) overall safer (3.10±0.80) than bushmeat species (2.59±0.02), where 1 = very dangerous, 2 = dangerous, 3 = neither safe nor dangerous, 4 = safe, 5 = very safe. Baboons (3.49±0.88), chimpanzees (3.48±0.89), goat (3.41±0.89), monkeys (3.38±0.94), pigs (3.32±0.90), and bats (3.28±0.94) were perceived by cooks to be the most likely to make a person sick when consumed, where 1 = very unlikely, 2 = unlikely, 3 = neither unlikely nor likely, 4 = likely, and 5 = very likely. Cooks identified edible bush rats as the least likely meat to make people sick when consumed (2.25±1.01), followed by beans and vegetables (2.32±0.98) and chicken (2.50±0.95). The perceived likelihoods that wildlife carried diseases that hunting dogs (3.53±0.89) or domestic livestock (3.54±0.85) could catch were comparable. Cutting and butchering meat during food preparation and active hunting were considered to carry the greatest risk of disease from wildlife (3.37±0.91) and (3.34±0.91) respectively, compared to trapping methods (3.08± 0.99). Cooking was perceived to carry notably less risk of disease than these activities (2.50±1.02). All above questions were scaled 1 = very unlikely, 2 = likely, 3 = neither unlikely nor likely, 4 = likely, 5 = very likely.

#### Species deception in market

**Fig 4:**
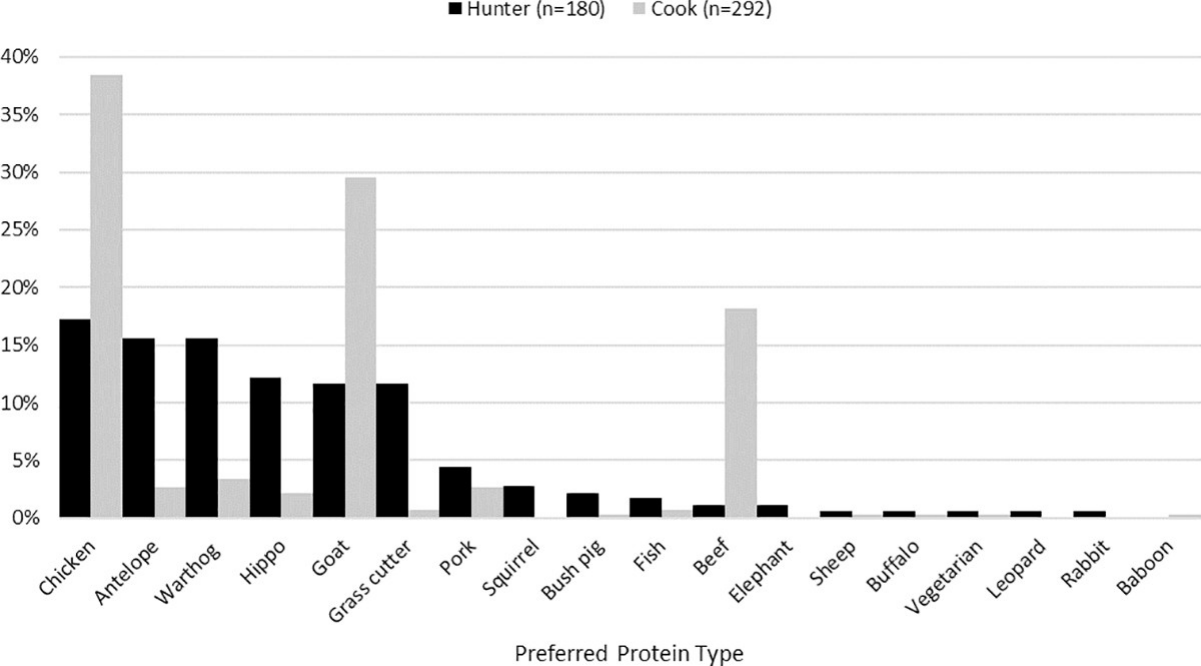
Cook and hunter responses to how often hunters and dealers disguise primate meat as another kind of meat to sell in Nwoya district, Uganda, 2016–2017. Independent t-tests show a significant difference in mean responses between cooks and hunters for both questions about how frequently hunters disguise primate meat (t_437.8_ = -35.3, p < 0.001) and how frequently dealers disguise primate meat (t_392.0_ = -63.3).

Species deception data for hunter and cooks are summarized in Fig 4. A notable majority of hunters (n = 156; 86.2%) report that they “usually” disguise primate meat as some other kind of meat in market. Furthermore, 95% (n = 172) of hunters report that dealers “usually” disguise primate meat as some other kind of meat in market. Cooks responded most frequently that they believed bushmeat hunters disguised primate (baboon, monkey, chimpanzee) as some other kind of meat to sell to never occur (n = 151; 50.2%), with virtually no cooks (n = 2; 0.7%) believing that it usually occurs. When asked how often market sellers or dealers disguise primate meat as some other kind of meat to sell, the majority of cooks again reported that this never happened (n = 255; 84.7%) and only one cook reported that they believed it usually occurs (n = 1; 0.3%); moreover, most cooks believe that baboons (n = 24l; 79.7%), monkeys (n = 250; 83.1%), chimpanzees (n = 264; 97.7%), and bats (n = 278: 92.4%) are “never” available in market to purchase. Independent t-tests confirm a significant difference in mean responses between cooks and hunters for both questions about hunters (t_437.8_ = -35.3, p < 0.001) and dealers (t_392.0_ = -63.3) disguising primate meat as another kind.

### 3.5 Perceived disease risk from bushmeat taxa

Principal components factor analysis results for the question “how likely it is that each wildlife species carry disease that humans can catch?” are summarized in Table 2. Both cooks’ and hunters’ responses grouped into 3 variables for these. Each animal was rated on a scale of 1–5 according to 1 = very unlikely, 2 = unlikely, 3 = neither unlikely nor likely, 4 = likely, and 5 = very likely; a lower number represents a perception of lower risk of contracting a zoonoses from that species/group. For cooks, primates (monkeys, baboons, chimpanzees) grouped together with the highest means (group $\bar{x}$ = 3.71), all domesticated animals eaten for meat grouped with the next highest means (group $\bar{x}$ = 3.43), and non-bat, non-primate wildlife grouped together for the lowest means (group $\bar{x}$ = 3.06). Bats did not fit into any of the factor reduction groupings for cooks ($\bar{x}$ = 3.41). For hunters, primates and bats grouped together (group $\bar{x}$ = 3.80), non-bat, non-primate wildlife species grouped together ($\bar{x}$ = 2.09), all domesticated animals (group $\bar{x}$ = 2.43), and edible bush rat did not group into any other factor ($\bar{x}$ = 1.63). Based on our threshold value of 0.5, porcupine was removed from the variable list for both hunters and cooks in the final analysis. Edible bush rats also fell below our threshold value for hunters and was removed from the final analysis.

**Table 2:** Principal components analysis with Varimax rotation of cook and hunter perceptions of zoonotic disease risk from various wildlife species, Uganda 2016–2017. Factor loadings above cutoff threshold of 0.5 are bolded in each column to indicate grouped factors. Columns for factor loadings were labeled with meaningful groups by authors based on representative group members.

Hunters (n = 180)						Cooks (n = 292)					
Animal type	$\bar{x}$	SE	Primates & Bats	Other wildlife	Domestic animals	Animal type	$\bar{x}$	SE	Primates	Other wildlife	Domestic animals
Baboon or monkey	3.99	0.092	**0.819**	0.139	0.063	Monkey	3.59	0.050	**0.778**	0.167	0.099
Bat	3.61	0.094	**0.732**	0.056	0.003	Baboon	3.75	0.042	**0.876**	0.100	0.156
Antelopes	1.68	0.081	-0.095	**0.732**	0.237	Chimpanzee	3.79	0.040	**0.909**	0.106	0.093
Buffaloes	2.33	0.105	0.076	**0.781**	0.076	Antelope	2.86	0.054	-0.010	**0.613**	0.295
Warthog or bushpig	2.54	0.105	0.291	**0.683**	0.068	Buffalo	3.17	0.053	0.162	**0.712**	0.160
Hippo	1.80	0.088	0.095	**0.769**	0.101	Bushpig	3.27	0.054	0.152	**0.865**	0.107
Cow	2.87	0.102	0.142	0.136	**0.773**	Warthog	3.17	0.055	0.178	**0.845**	0.0646
Chicken	2.12	0.102	0.003	-0.006	**0.716**	Edible bushrat	2.82	0.055	0.045	**0.586**	0.317
Goat	2.31	0.103	-0.056	0.288	**0.689**	Cow	3.62	0.050	0.328	0.129	**0.598**
						Chicken	3.32	0.059	-0.030	0.142	**0.734**
						Pig	3.63	0.045	0.119	0.184	**0.760**
						Goat	3.16	0.056	0.133	0.257	**0.751**
Eigenvalues			1.136	2.896	1.338	Eigenvalues			1.823	4.414	1.369
Variance explained (%)			12.62	32.17	14.87	Variance explained (%)			15.19	36.78	11.41
Cronbach's α			0.444	0.749	0.583	Cronbach's α			0.842	0.816	0.739

## 4. Discussion

The findings of this study emphasize important areas of concern for public health measures from the bushmeat trade in northern Uganda. Most of our respondents in both hunter and cook surveys reported their primary occupation as farming, which is consistent with other studies in sub-Saharan Africa where hunting is seen as supplemental to agricultural activities rather than a primary occupation [64–68]. Bushmeat hunting is thought to be primarily done as a source of supplemental income or to ensure household food security. Interviews of UWA law enforcement officers in Queen Elizabeth National Park corroborate the need for bushmeat for both personal consumption and generation of basic income, citing poverty and lack of economic opportunity as the main reasons for poaching [44]. Still, our findings indicate that preference for wild animal meat may play a role in bushmeat utilization, consistent with similar studies, as four of the five top preferred meats by hunters were wild animals rather than domestic choices [69–71]. This finding is not mirrored by the reported preferences of cooks, who generally preferred domestic meat options and believed domestic meat choices to be more nutritious than bushmeat, which may indicate that male household members may have more influence over household food choices.

Based on responses to our questions about diseases that wildlife carry, almost all respondents were aware that there is a real and present risk of disease spillover from wildlife to people. Epidemics in recent years may contribute to this knowledge, but for hunters this awareness does not appear to influence or motivate any precautionary behaviors during the harvest of wildlife as virtually no respondents reported taking precautions. Rather, the precautions that were reported were related to the potential for legal or financial repercussions if caught by authorities for poaching. The most reported precaution was “butchering in the field” and “leaving the bones behind” to minimize evidence of poaching. Similar to studies in Central Africa, these responses suggest that risk of illness or injury from bushmeat hunting does not outweigh the incentive of financial profit from the sale or use value of the harvested bushmeat [72].

Previous research has shown that there is nearly a 30% discrepancy between what species bushmeat is being sold as by hunters and dealers and what species are actually being sold in Uganda (Dell, in review). The data in this paper substantiate that this deception may be intentional by hunters in many cases. Most hunters interviewed reported that they usually disguised primate meat as another species and that they knew dealers of bushmeat would often do the same; however, cooks’ responses to the same question indicate they do not believe that this deception occurs. Although only disguising primates was asked about in our surveys, data form Dell et al. reveal that this intentional deception is not restricted to species that are taboo to consume and includes the disguise of species that were most preferred in this study as other kinds of bushmeat. This incongruity is potentially harmful because it subverts the ability of bushmeat consumers to make informed choices about their diets. Moreover, the way that cooks responded to the question about diseases humans can catch from wildlife indicates that there is awareness that certain species carry more inherent risk for zoonoses transmission than others. If we assume that this translates to differences in precautionary practices in food preparation and handling, then consumers may be inadvertently exposing themselves and others consuming the meals to zoonotic pathogens due to this misrepresentation. Most cooks we interviewed noted that they did not eat bats and primates; this should thereby confer a degree of ‘cultural immunity’. The phenomenon of market deception and hunters admitting to eating bats and primates in the bush may challenge the degree that preference and choice protect community members from exposure to zoonotic pathogens carried by species with a high risk of spillover.

Hunters have arguably the greatest amount of contact with animal tissue through the process of hunting itself. Even with snares and traps, the risk of injury during these events is high, particularly if the animals are not found dead when the traps are checked, and the wounded animal must be killed at close range. Inhalation of aerosolized particles on fur or urine of wildlife, inadvertent fecal-oral transmission when handling the carcass, bloodborne transmission during the killing and butchering process, as well as the potential for transmission through saliva via a bite during the kill all pose serious threats to the health of hunters [73]. Although the majority of hunters did not report frequently being injured during hunting, trapping, and butchering, multiple hunters did admit to butchering wildlife carcasses hastily in the field to leave behind the bones which may reasonably lead to increased incidence of injury. Injury remains a common experience as part of bushmeat harvest, with incidence of injury to bushmeat hunters in a community in western Uganda at over 13% and nearly 60% of those injured seeking medical care for their injuries [74]. Hunting using firearms may reduce contact with live animals if hunters are accurate shots, however, civilian-owned firearms in Uganda are strictly regulated through fire-arm certificates and stringently enforced. We did not ask about hunting with firearms on the advice of our colleagues in Uganda. The sensitive nature of this subject led us to believe that self-reporting of use would be inaccurate or discourage study participation. Although it is not reported in our study, hunting with firearms is common in other areas of sub-Saharan Africa [67,75–77].

Hunters most reported trapping using wire neck- or leg-hold snares. This and the other non-selective hunting measures most frequently reported in our study are consistent with commonly used methods across the tropics and subtropics for their relative ease of use, but pose a particular threat to wildlife [78,79]. Non-selective hunting methods result in substantial bycatch of non-target species which leads to decomposition or scavenging, may disproportionately impact threatened species, and may result in intentional wasting if traps are inconveniently located to hunters or if less profitable species are snared [79–81]. This practice poses a threat to the sustainability of wildlife populations, particularly wildlife populations in border zones of these protected areas where human populations are dense and access to protected areas is convenient. In this study, wasting due to capture of non-target species may be less of an issue since hunters reported bringing back meat that was already butchered in the field and is presumably more likely to be passed off as more in-demand meats or meats that will fetch a higher market price [45].

In both hunter and cook groups, primates were considered to present a higher risk of zoonotic disease transmission than other species. For hunters, bats grouped with primates as the highest-risk species. Cooks responses grouped primates together as the highest-risk species, but bats did not group with them and had a lower mean response. This difference may be explained by the fact that many women married into the community and may have come from nearby mountainous regions where bats are more often consumed and are not considered a high-risk animal for disease spillover (Dell and Willcox Personal Communications). During interviews, both cooks and hunters indicated that in the more mountainous regions nearby, larger bat species are commonly consumed, whereas in Nwoya district, most did not report that they considered bats edible or a preferred species (Dell and Willcox Personal Communications). Cooks considered domesticated animals, rather than wildlife, to have the next greatest zoonotic risk, where hunters considered what broadly grouped as other wildlife to have the next greatest zoonotic risk. Veterinary outreach efforts to promote vaccination and domestic animal health in Nwoya district historically tended to target the women in the household, as livestock rearing and farming is typically their responsibility (Dell Personal Communications). This increased awareness of domestic animal health and disease may contribute to cooks’ responses, indicating that educational campaigns may be an effective strategy for mitigating food-related infections.

Few cooks reported taking special precautions when preparing either bushmeat or domestic meat. Moreover, a greater proportion of cooks reported taking precautions when handling domestic meat than bushmeat. This is consistent with the belief that domestic species are more likely to cause disease in people than most wildlife. Cooks responses indicated that although most of them have a level of concern about diseases from bushmeat at the time of purchase, that concern decreases during cooking/preparation, and decreases even further at the time of consumption. This finding either speaks to confidence in appropriate food safety technique or is an example of awareness of an abstract issue, like emerging zoonotic diseases, that has little relevance to them on a practical and day to day level.

The complexity of the issue of bushmeat presents challenges to efforts to adapt data about the practice into useful and practical intervention strategies. Engaging our target population involves communicating that the risk of zoonotic disease spillover and threats to conservation are both relevant and of consequence to them specifically. Even if this is achieved, evidence to support awareness and concern as adequate motivation to elicit behavioral changes, especially when these changes are impractical or costly, is not well supported [72,82]. Further data suggest that intervention strategies that depend on informal societal mores and local-level institutions may have greater buy-in than governmental level regulations [83,84].

It is important to consider that hunting in our study area remains an illicit activity and the threat of discovery or implication of participation in poaching may have deterred participation of both hunters and cooks. The illegality of hunting may have also biased responses of those who participated in the study, leading to underestimations of participation. Additionally, questions about disguising meat as another kind may have bias in responses, as cooks acknowledging that this occurs directly implicates members or their communities in deceptive behavior. Similarly, responses by cooks about preferred meat choices may underrepresent a preference for bushmeat, due to issues surrounding its legality.

## 5. Conclusions

We have provided important insights into awareness of zoonoses and occupational injury for community members involved in the bushmeat commodity chain, as well as patterns of meat preference among hunters and cooks in villages bordering MFCA. These data clarify points in the bushmeat commodity chain, namely butchering, trapping, and contact with incorrectly specified bushmeat tissue, where cooks and hunters are most susceptible to injury and exposure to infectious agents. More detailed evaluations of subjective cultural characteristics of this community, such as beliefs, attitudes, and social norms of the community as a whole rather than hunters and cooks alone, will help in understanding determinants, practices, and preferences in the bushmeat trade. This will ultimately lead to the development of more successful and appropriate conservation tactics for wildlife species in MFNP. Furthermore, increasing community engagement and advancing community understanding of the interplay between wildlife species and their own health may inform approaches by public health entities that ultimately increase the communities perceived control of mitigating their own disease risk.

## Supporting information

S1 File Survey instruments used to interview hunters and women who cook in Nwoya district, Uganda, 2016. (DOCX)

S2 File Survey instruments used to interview hunters and women who cook in Nwoya district, Uganda, 2017. (DOCX)
