# Correction: Attitudes, practices, and zoonoses awareness of community members involved in the bushmeat trade near Murchison Falls National Park, northern Uganda

**DOI:** 10.1371/journal.pone.0325743

**Published:** 2025-05-29

**Authors:** BreeAnna M. Dell, Marcy J. Souza, Adam S. Willcox

The images for Figs 2 and 4 are incorrectly switched. The image that appears as Fig 2 should be Fig 4, and the image that appears as Fig 4 should be Fig 2. The figure captions appear in the correct order. Please see the images and captions in the correct order below.

**Fig 2:**
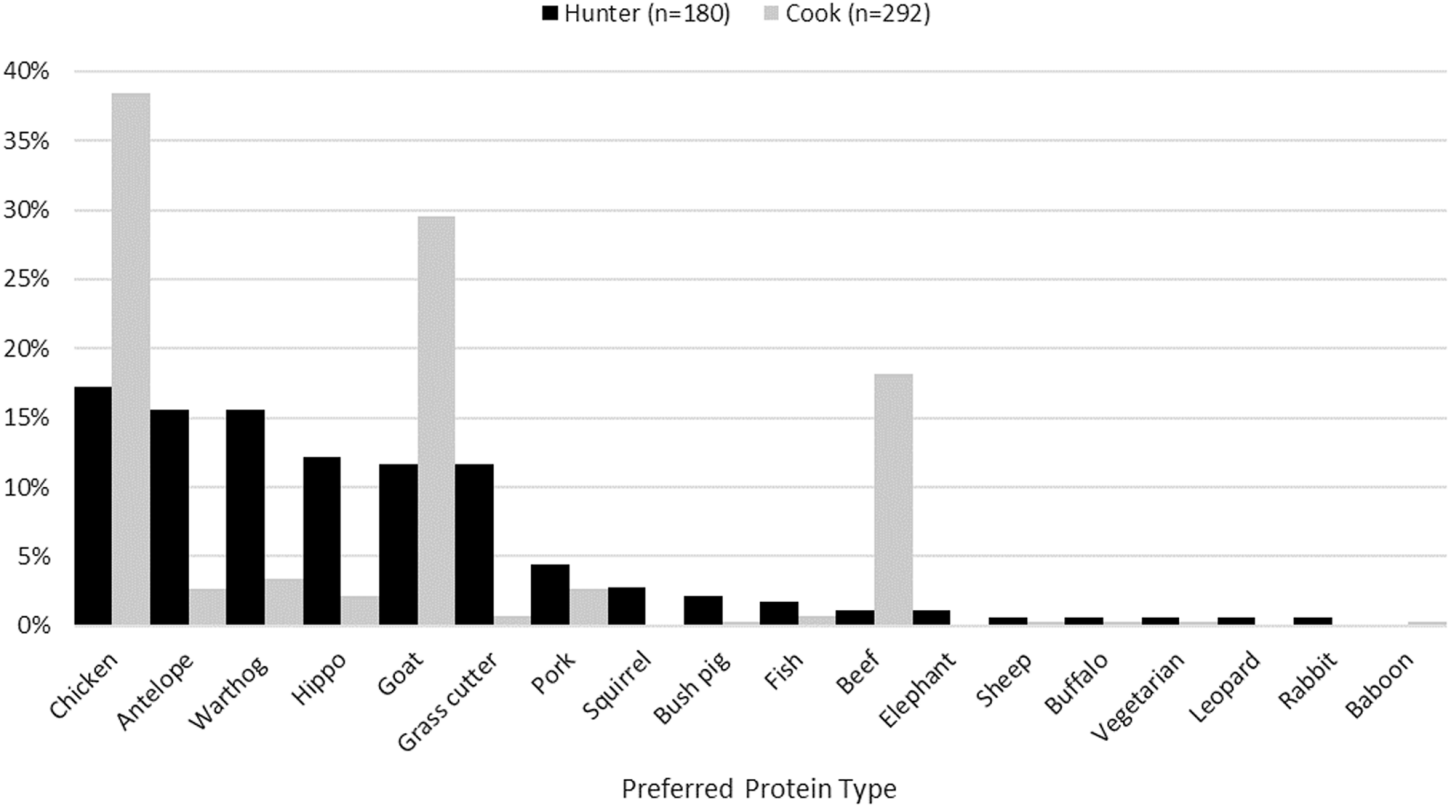
Cook and hunter responses to which type of meat they most prefer to eat from among wild and domestic choices in Nwoya district, Uganda, 2016–2017.

**Fig 4:**
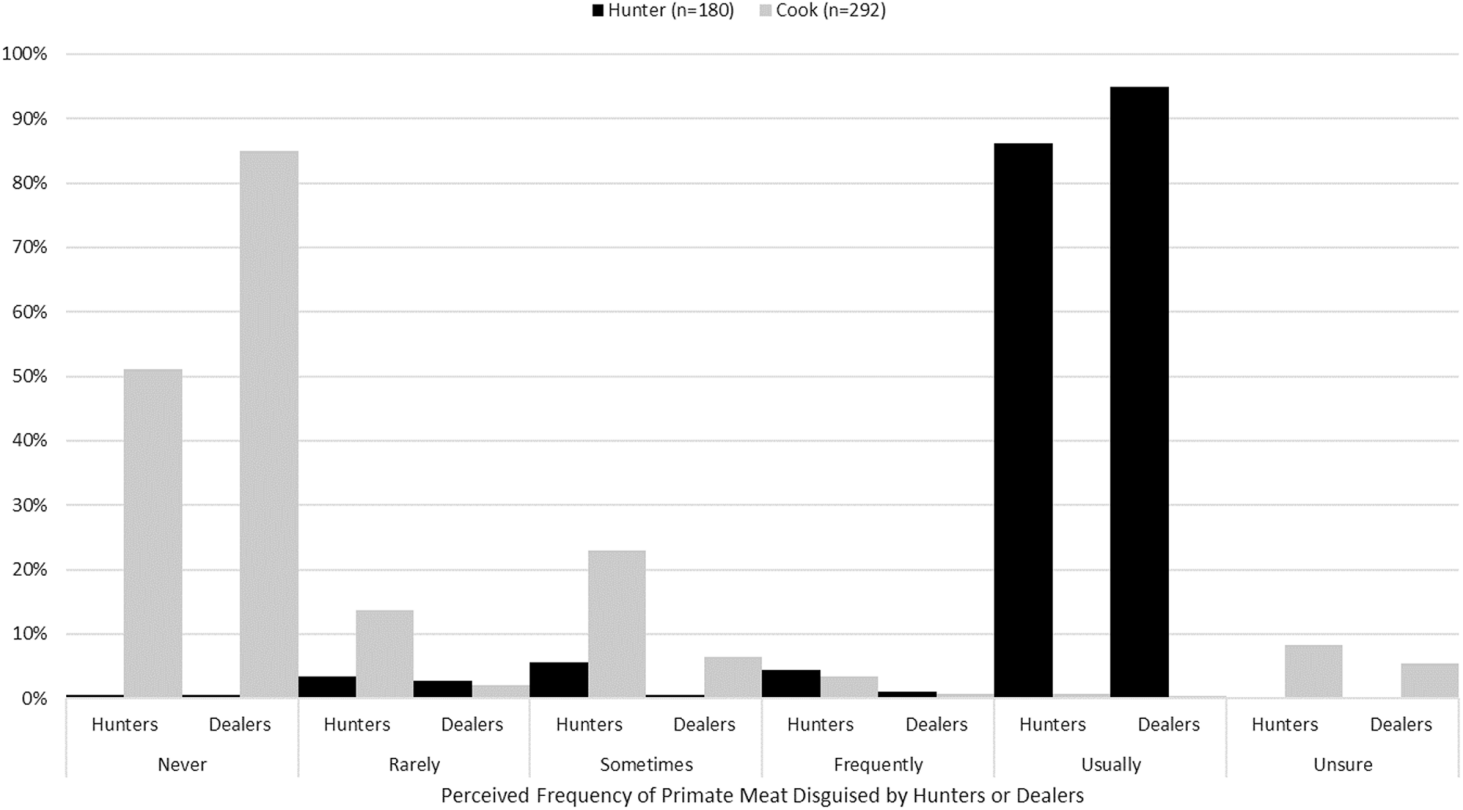
Cook and hunter responses to how often hunters and dealers disguise primate meat as another kind of meat to sell in Nwoya district, Uganda, 2016–2017. Independent t-tests show a significant difference in mean responses between cooks and hunters for both questions about how frequently hunters disguise primate meat (t_437.8_ = −35.3, p < 0.001) and how frequently dealers disguise primate meat (t_392.0_ = −63.3).
