# Supplementary material for: Attitudes, practices, and zoonoses awareness of community members involved in the bushmeat trade near Murchison Falls National Park, northern Uganda
Source: PLoS One. 2020 Sep 28;15(9):e0239599. doi: 10.1371/journal.pone.0239599 (PMC7521682; doi:10.1371/journal.pone.0239599)
Supplement: S1 File — (DOCX) [file pone.0239599.s001.docx]

**Cook Survey Instrument**

Description:

Introduction: Makerere University Department of Zoology, Entomology, and Fisheries Science and the University of Tennessee's Department of Forestry, Wildlife and Fisheries and College of Veterinary Medicine are conducting research to learn what hunters think about wildlife and diseases. Thank you for volunteering your help!

INFORMATION ABOUT PARTICIPANTS' INVOLVEMENT IN STUDY The questions in this survey ask you about preparing meat for food, what you think about the potential to catch diseases from wildlife, and finally some basic information about you. No personal identifying information will be collected. You must be 18 years of age or older to participate. The survey should take about 10-15 minutes to complete.

Conclusion: Thank you for completing the survey!

Q 1 Information

RISKS There are no foreseeable risks other than those encountered in everyday life.

BENEFITS Results from this research will help Ugandans make informed healthy choices about bush meat consumption.

CONFIDENTIALITY The information in the study records will be kept confidential. Data will be stored securely and will be made available only to persons conducting the study unless participants specifically give permission in writing to do otherwise. No reference will be made in oral or written reports which could link participants to the study.

CONTACT INFORMATION If you have questions at any time about the study or the procedure (or you experience adverse effects as a result of participating in the study), you may contact the researcher, Dr. BreeAnna Dell, at yjf729@vols.utk.edu. If you have questions about your

rights as a participant, you may contact the University of Tennessee IRB Compliance Officer at utkirb@utk.edu or (865) 974-7697.

PARTICIPATION Your participation in this study is voluntary; you may decline to participate without penalty. If you decide to participate, you may withdraw from the study at any time without penalty. If you withdraw from the study before data collection is completed, your data will be permanently deleted.

CONSENT I have read the above information. I have received (or had the opportunity to print) a copy of this form. Clicking on the button to continue and completing the survey constitutes my consent to participate.

Q 2

What is the most delicious meat?

1 Chicken

2 Fish

3 Beef

4 Goat

5 Pork

6 Rabbit

7 Pigeon

8 Antelope

9 Edible bush rodent (grass cutter)

10 Porcupine

11 Bush pig

12 Warthog

13 Chimpanzee

14 Baboon

15 Monkey

16 Elephant

17 Buffalo

18 Giraffe

19 Crocodile

20 Hippo

21 Other

Q 3

What is the most delicious wild meat?

1 Edible bush rodent (grass cutter)

2 Porcupine

3 Antelope

4 Bush pig

5 Warthog

6 Baboon

7 Monkey

8 Giraffe

9 Elephant

10 Buffalo

11 Crocodile

12 Hippo

13 Other

Q 4

What is the most delicious domestic animal?

1 Cow

2 Chicken

3 Rabbit

4 Pigeon

5 Pig

6 Goat

7 Sheep

8 Other

Q 5

What is the most nutritious meat?

1 Cow

2 Bushmeat

3 Chicken

4 Fish

5 Pig

6 Goat

7 Sheep

Q 6

How expensive is bushmeat?

1 Very cheap

2 Cheap

3 Neither cheap nor expensive

4 Expensive

5 Very expensive

Q 7

How expensive is domestic meat (cow, pig, chicken, goat, sheep)?

1 Very cheap

2 Cheap

3 Neither cheap nor expensive

4 Expensive

5 Very expensive

Q 8

What do you like better to eat?

1 Fish

2 Meat

3 Beans/vegetables

Q 9

How safe is it to consume bushmeat?

1 Very dangerous

2 Dangerous

3 Neither safe nor dangerous

4 Safe

5 Very safe

Q 10

How safe is it to consume domestic meat (cow, pig, chicken)?

1 Very dangerous

2 Dangerous

3 Neither dangerous nor safe

4 Safe

5 Very safe

Q 11

Which diseases do wildlife carry?

Statements

S 1 Marburg virus

S 2 Ebola

S 3 African swine fever

S 4 Stomach ache or diarrhoea

S 5 Malaria

S 6 Monkeypox

S 7 Plague

S 8 Brucellosis

S 9 Scabies

Answers

A 1 Yes

A 2 No

A 3 I don't know

Q 12

How likely is it that each of these animals carry diseases that humans could catch?

Statements

S 1 Monkeys

S 2 Baboons

S 3 Chimpanzees

S 4 Bats

S 5 Antelopes (all kinds)

S 6 Buffaloes

S 7 Bush pigs

S 8 Wart hogs

S 9 Edible bush rats (grass cutters)

S 10 Porcupine

S 11 Cow

S 12 Chicken

S 13 Pig

S 14 Goat

S 15 Sheep

Answers

A 1 Very unlikely

A 2 Unlikely

A 3 Neither unlikely nor likely

A 4 Likely

A 5 Very likely

Q 13

How likely is it that wildlife carry diseases that hunting dogs could catch?

1 Very unlikely

2 Unlikely

3 Neither unlikely nor likely

4 Likely

5 Very likely

Q 14

How likely is it that wildlife carry diseases that livestock could catch?

1 Very unlikely

2 Unlikely

3 Neither unlikely nor likely

4 Likely

5 Very likely

Q 15

How likely is it that people could get sick from wildlife while hunting?

1 Very unlikely

2 Unlikely

3 Neither unlikely nor likely

4 Likely

5 Very likely

Q 16

How likely is it that people could get sick from wildlife while trapping?

1 Very unlikely

2 Unlikely

3 Neither unlikely nor likely

4 Likely

5 Very likely

Q 17

How likely is it that people could get sick from wildlife while cutting/butchering?

1 Very unlikely

2 Unlikely

3 Neither unlikely nor likely

4 Likely

5 Very likely

Q 18

How likely is it that people could get sick from wildlife while cooking it?

1 Very unlikely

2 Likely

3 Neither unlikely nor likely

4 Likely

5 Very likely

Q 19

How often do you get a wound when preparing or cooking meat?

1 Never

2 Rarely

3 Sometimes

4 Frequently

5 Usually

Q 20

Do you take any special precautions when cooking or preparing bushmeat?

1 Yes

2 No

Q 21

What precautions do you take?

Q 22

Do you take special precautions when you prepare or cook meat from domestic animals

(cow, pig, chicken, goat, sheep)?

1 Yes

2 No

Q 23

What precautions do you take?

Q 24

How likely is it that people could get sick from

eating each of the following?

Statements

S 1 Monkeys

S 2 Baboons

S 3 Chimpanzees

S 4 Bats

S 5 Antelopes

S 6 Buffaloes

S 7 Bushpigs

S 8 Wart hogs

S 9 Porcupine

S 10 Hippo

S 11 Giraffe

S 12 Chicken

S 13 Cows (beef)

S 14 Pigs (pork)

S 15 Goat

S 16 Sheep

S 17 Fish

S 18 Beans/vegetables

S 19 Edible bush rats (grass cutter)

Answers

A 1 Very unlikely

A 2 Unlikely

A 3 Neither unlikely nor likely

A 4 Likely

A 5 Very likely

Q 25

How often do you think about catching diseases from bushmeat when buying meat to

cook?

1 Never

2 Rarely

3 Sometimes

4 Frequently

5 Every time

Q 26

How often do you think about catching diseases from bushmeat when butchering or

cooking meat?

1 Never

2 Rarely

3 Sometimes

4 Frequently

5 Every time

Q 27

How often to you think about catching diseases from eating bushmeat?

1 Never

2 Rarely

3 Sometimes

4 Frequently

5 Every time

Q 28

How frequently do you eat each of the following?

Statements:

S 1 Baboons

S 2 Monkeys

S 3 Chimpanzees

S 4 Bats

Answers:

A 1 Never

A 2 Rarely

A 3 Sometimes

A 4 Frequently

A 5 Usually

A 6 Every day

Q 29

How often is there baboon meat available to purchase?

1 Never

2 Rarely

3 Sometimes

4 Frequently

5 Usually

6 Every time

Q 30

How often is there monkey meat available to purchase?

1 Never

2 Rarely

3 Sometimes

4 Frequently

5 Usually

6 Every time

Q 31

How often is there chimpanzee meat available to purchase?

1 Never

2 Rarely

3 Sometimes

4 Frequently

5 Usually

6 Every time

Q 32

How often is there bat meat available to purchase?

1 Never

2 Rarely

3 Sometimes

4 Frequently

5 Usually

6 Every time

Q 33

How often do hunters disguise baboon, chimpanzee, or monkey meat as some other

meat?

1 Never

2 Rarely

3 Sometimes

4 Frequently

5 Usually

6 I don't know/no opinion

Q 34

How often do market sellers disguise baboon, chimpanzee, or monkey meat as some other

meat?

1 Never

2 Rarely

3 Sometimes

4 Frequently

5 Usually

6 I don't know/no opinion

Q 35

What year were you born?

Q 36

How long have you lived in this community (in years)?

Q 37

What was your last level of school education?

1 Primary school

2 Secondary school

3 College or university

4 Technical/trade school

5 Graduate school

6 Informal/no formal schooling

Q 38

What is your primary occupation?

Q 39

Are you:

1 Married

2 Divorced

3 Widow

4 Never married

Q 40

How many adults do you usually cook for?

Q 41

How many children do you usually cook for?
