# Supplementary material for: Attitudes, practices, and zoonoses awareness of community members involved in the bushmeat trade near Murchison Falls National Park, northern Uganda
Source: PLoS One. 2020 Sep 28;15(9):e0239599. doi: 10.1371/journal.pone.0239599 (PMC7521682; doi:10.1371/journal.pone.0239599)
Supplement: S2 File — (DOCX) [file pone.0239599.s002.docx]

Hunter Survey Instrument

Introduction: Makerere University Department of Zoology, Entomology and Fisheries Sciences and the University of Tennessee’s Department of Forestry, Wildlife and Fisheries and College of Veterinary Medicine are conducting research to learn what hunters think about wildlife and diseases. Thank you for volunteering your help!

INFORMATION ABOUT PARTICIPANTS’ INVOLVEMENT IN STUDY The questions in this survey ask you about hunting, what you think about the potential to catch diseases from wildlife, and finally some basic information about you. No personal identifying information will be collected. You must be 18 years of age or older to participate. The survey should take about 10-15 minutes to complete.

Conclusion: Thank you for completing the survey

Q1

RISKS There are no foreseeable risks other than those encountered in everyday life.

BENEFITS Results from this research study will help Ugandans make informed healthy choices about bushmeat consumption.

CONFIDENTIALITY The information in the study records will be kept confidential. Data will be stored securely and will be made available only to persons conducting the study unless participants specifically give permission in writing to do otherwise. No reference will be made in oral or written reports which could link participants to the study. CONTACT INFORMATION If you have questions at any time about the study or the procedures (or you experience adverse effects as a result of participating in this study), you may contact the researcher, Dr. Adam Willcox, at awillcox@utk.edu or (865) 9741557. If you have questions about your rights as a participant, you may contact the University of Tennessee IRB Compliance Officer at utkirb@utk.edu or (865) 9747697.

PARTICIPATION Your participation in this study is voluntary; you may decline to participate without penalty. If you decide to participate, you may withdraw from the study at any time without penalty. If you withdraw from the study before data collection is completed your data will be permanently deleted.

CONSENT I have read the above information. I have received (or had the opportunity to print) a copy of this form. Clicking on the button to continue and completing the survey constitutes my consent to participate.

Q 2

What is the most delicious meat?

1 Chicken

2 Fish

3 Beef

4 Goat

5 Sheep

6 Pork

7 Edible bush rat (grass cutter)

8 Porcupine

9 Bush pig

10 Warthog

11 Baboon

12 Monkey

13 Buffalo

14 Hippo

15 Other

Q 3

What is the most delicious wild meat?

1 Edible bush rat (grass cutter)

2 Porcupine

3 Antelope

4 Bush pig

5 Warthog

6 Baboon

7 Monkey

8 Buffalo

9 Hippo

10 Other

Q 4

What is the most delicious domestic animal?

1 Cow

2 Chicken

3 Goat

4 Sheep

5 Pig

6 Other

Q 5

What do you like better to eat?

1 Meat

2 Fish

3 Beans/vegetables

Q 6

What is the most dangerous animal to hunt?

Q 7

What is the most dangerous animal to trap?

Q 8

How safe or dangerous are the following:

Answers

A 1 Very Safe

A 2 Safe

A 3 Neither Safe nor Dangerous

A 4 Dangerous

A 5 Very Dangerous

Statements

S 1 catching animals with a trap.

S 2 hunting animals with a spear

S 3 hunting animals with dogs

S 4 hunting animals with bow and arrow

Q 9

How often do you hunt using the following methods:

Answers

A 1 Nearly every day

A 2 At least 3 times per week

A 3 Once a week

A 4 Several times per month

A 5 Several times per year

A 6 Never

Statements

S 1 Wire snare

S 2 Leghold spring trap

S 3 Dogs

S 4 Pitfall

S 5 Net

S 6 Spear

S 7 Stick/club

S 8 bow and arrow

Q 10

How often do you get a wound when:

Answers

A 1 Never

A 2 Rarely

A 3 Sometimes

A 4 Frequenty

A 5 Every time

A 6 Not applicable, I do not do this

activity

Statements

S 1 Hunting with a spear

S 2 Trapping (all kinds)

S 3 Cutting/butchering meat

S 4 Hunting with dogs

Q 11

Which of these diseases do wildlife carry?

Answers

A 1 Yes

A 2 No

A 3 Don't know

Statements

S 1 Marburg

S 2 Ebola

S 3 African swine fever

S 4 Malaria

S 5 Stomach ache or diarrhoea

S 6 Pox

S 7 Brucellosis

S 8 Scabies

Q 12

How likely is it that the following animals carry diseases that humans can catch?

Answers

A 1 Very unlikely

A 2 Unlikley

A 3 Neither unlikely or likely

A 4 Likley

A 5 Very likely

Statements

S 1 Baboons or monkeys

S 2 Bats

S 3 Antelopes (all kinds)

S 4 Buffaloes

S 5 Warthogs or bushpigs

S 6 Hippos

S 7 Edible bush rat (grass cutter)

S 8 Porcupine

S 9 Cow

S 10 Chicken

S 11 Fish

S 12 Goat

Q 13

How likely is it that wildlife carry diseases that could be transferred to:

Answers

A 1 Very unlikely

A 2 Unlikely

A 3 Neither unlikely nor likely

A 4 Likely

A 5 Very likely

Statements

S 1 hunting dogs

S 2 livestock

S 3 people

Q 14

How likely is it that people could get sick from:

Answers

A 1 Very unlikely

A 2 Unlikely

A 3 Neither unlikely of likely

A 4 Likely

A 5 Very likely

Statements

S 1 hunting? (from the act of hunting)

S 2 trapping? (from the act of trapping)

S 3 butchering, cutting, or preparing

wildlife/bushmeat?

S 4 eating baboons or monkeys?

S 5 eating bats?

S 6 eating antelopes (all kinds)?

S 7 eating buffaloes?

S 8 eating warthogs or bush pigs?

S 9 eating hippos?

S 10 eating edible bush rat (grass

cutter)?

S 11 eating porcupines?

S 12 eating chicken?

S 13 eating cows (beef)?

S 14 eating pork?

S 15 eating fish?

S 16 eating beans and vegetables?

Q 15

Have you ever harvested, hunted, or trapped:

Answers

A 1 Yes

A 2 No

Statements

S 1 baboons?

S 2 monkeys?

S 3 bats?

Q 16

How often are the following animals available to purchase from dealers

Answers

A 1 Every week

A 2 Several times per month

A 3 Several times a year

A 4 Every few years

A 5 Never

Statements

S 1 baboons

S 2 monkeys

S 3 bats

S 4 antelopes (all kinds)

S 5 edible bush rats (cutting grass)

S 6 hippos

S 7 buffaloes

S 8 bush pigs and wart hogs

S 9 porcupines

Q 17

How often do hunters disguise baboon or monkey meat as some other meat?

1 Never

2 Rarely

3 Sometimes

4 Frequently

5 Usually

Q 18

How often do dealers disguise baboon or monkey meat as some other meat?

1 Never

2 Rarely

3 Sometimes

4 Frequently

5 Usually

Q 19

Do you take any special precautions when hunting, trapping, or handling bushmeat?

1 Yes

2 No

Q 20

What special precautions do you take?

Q 21

How old are you?

Q 22

How long have you lived in this community?

1 Since birth

2 1-5 years

3 6-10 years

4 11-20 years

5 20+ years

Q 23

What is your last level of school education?

1 Primary school

2 Secondary school

3 College or university

4 Technical/trade school

5 Graduate school

Q 24

What is your primary occupation?

Q 25

Are you:

1 Married

2 Single (never married)

3 Divorced

4 Widower
